# Colonic carcinoma with multiple small bowel perforations mimicking intestinal obstruction

**DOI:** 10.1186/1477-7819-4-63

**Published:** 2006-09-15

**Authors:** Satyendra K Tiwary, Manish K Singh, Rahul Khanna, Ajay K Khanna

**Affiliations:** 1 Department of General surgery, Institute of Medical Sciences, Banaras Hindu University, Varanasi-221005, India

## Abstract

**Background:**

Carcinoma of the colon may present with perforation proximal to the site of malignancy. Caecum is the commonest site of perforation if the ileocecal valve is patent and the jejunal and ileal perforations are very rare.

**Case presentation:**

A 35 year male presented with intestinal obstruction. Emergency laparotomy revealed carcinoma of the transverse colon with multiple pinpoint perforations along antimesenteric border of ileum, which were wrapped with omentum, and no peritoneal contamination was present. Extended right hemicolectomy with jejunocolic anastomosis was done. Patient made uneventful recovery in postoperative period and was treated with adjuvant chemotherapy.

**Conclusion:**

Patients with colonic carcinoma and incompetent ileocecal valve may present with intestinal perforation. Increased intraluminal pressure and closed loop obstruction may lead to ischemia and perforation of the small bowel.

## Background

It is very unusual to find a case of carcinoma colon with associated multiple perforations of proximal part. In 4 to 5% cases, presentation of carcinoma colon is with perforation. In these cases high mortality has been reported in literature from 30 to 40% [1]. Perforation may occur if tumor invades serosal layer, but this perforation is usually solitary and large. Peritoneal contamination is invariably associated. We hereby report a case of carcinoma of transverse colon presenting with acute intestinal obstruction. Laparotomy revealed carcinoma of transverse colon with dilated small bowel and multiple small pin point perforations along antimesenteric border wrapped up by omentum. There was no peritoneal contamination and patient recovered without any post operative complication.

## Case presentation

A 35 year male presented with features of acute intestinal obstruction. He was unable to pass faeces and flatus with pain in abdomen since last five days. On examination abdomen was distended, shifting dullness was absent, and the bowel sounds were absent. Patient gave a history of on and off abdominal pain and occasional melena during last ten months.

Biochemical parameters revealed decreased hemoglobin (8.2 gm/dl). Other parameters were within normal limits. X-ray of abdomen in erect posture revealed multiple air fluid level and dilated small bowel loops. There was no free gas under the diaphragm. Ultrasound of abdomen revealed dilated small intestine loops suggestive of sub acute intestinal obstruction.

With a provisional diagnosis of intestinal obstruction, exploratory laparotomy was planned. On opening peritoneum, dilated jejunum with omentum wrapped along the antimesenteric border of ileum was found. There was no free fluid in peritoneal cavity. On separating omentum from ileum, multiple pinpoint perforations along the antimesenteric border were noted. Ileum was dilated with multiple patchy gangrenous regions with picture suggestive of ischemic enteritis. A 4 × 3 cm mass occupying transverse colon just distal to hepatic flexure of colon was also found. There were two metastatic nodules on superior surface of right lobe of liver. Extended hemicolectomy was done with resection of ischemic and perforated ileum, cecum, appendix, ascending colon, transeverse colon (proximal two third) and colo-enteric anastomosis was performed. After putting drain in the pelvis, abdomen was closed.

**Figure 1:**
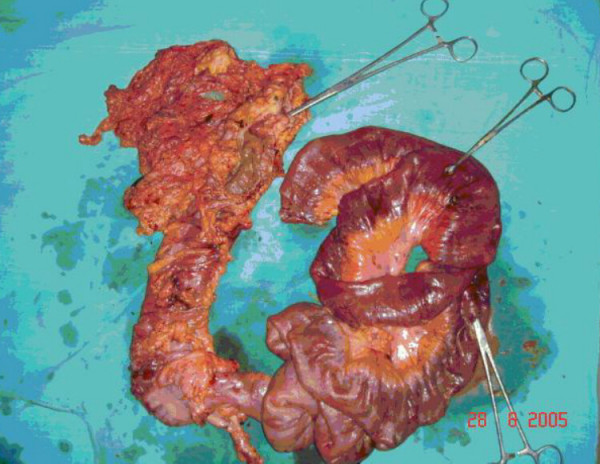
Photograph of resected bowel with ileal perforations and carcinoma colon.

**Figure 2:**
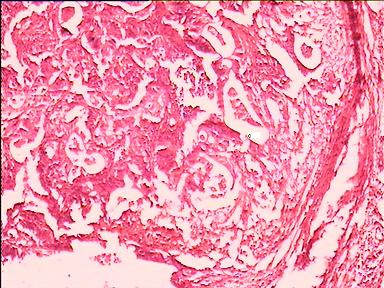
Microphotograph of adenocarcinoma colon (H & E × 500).

Gross examination of the resected segment revealed ischemic ileum about 4 feet in length with multiple perforations along antimesenteric border (Figure 1). Cecum, appendix and ascending colon did not show any obvious lesion. An ulceroproliferative lesion of 2 cm × 2 cm was present in longitudinally opened resected transverse colon. Histopathological examination confirmed moderately differentiated adenocarcinoma (Figure 2) involving serosa with ischemia and necrosis of ileum. Subsequently, chemotherapy was planned.

## Discussion

Colon carcinoma may present with perforations causing generalized peritonitis or localized peritonitis. Perforation usually occurs at the site of tumor due to its growth and pressure necrosis of walls. Solitary perforations are usual presentations. In difficult colonoscopy due to stricture perforation may occur proximally in cecum due to pneumatic dilatation [2]. Endoluminal hypertension-ischemia is usually responsible for perforations in colonic carcinoma but in cases with non-occluded colon, as a consequence of biological problems of immune hyper reactivity of a rejection reaction type leads to perforation [3]. Bacterial and viral overgrowth in obstructed and ischemic bowel may lead to increased intraluminal pressure causing ischemic enteritis and subsequent perforation of dilated small bowel if ileocecal valve is incompetent or malfunctioning.

One notable thing observed in this case was incompetent ileocecal valve preventing closed loop obstruction of colon and transmitting backpressure in ileum causing marked dilatation and ischemic enteritis leading to multiple perforations. In spite of serosal involvement, no perforation was present at the site of tumor. No ascites was present despite hepatic metastasis. Pericolic abscess may be the presentation in perforations if it is localized and sealed by omentum. In our case no pericolic abscess was present as multiple pinpoint perforations were wrapped up adequately with omentum preventing any peritoneal contamination required for localized or generalized peritonitis.

Clinical diagnosis of such complications of colonic malignancy is impossible. Even endoscopies or barium study are unlikely to reveal pinpoint ileal perforations. Such cases of malignancy are most likely to be managed by emergency exploratory laparotomy. Decision of procedure to be performed is likely to be taken intraoperatively influenced by findings on opening peritoneal cavity. These cases present with obstruction despite perforations being present. Primary resection with anastomosis is the procedure of choice in obstructing lesions of the right colon. This has a lower operative mortality and morbidity than a staged procedure. This primary resection with anastomosis is certainly as safe as an ileotransverse colostomy[4].

Colonic carcinoma with perforations and peritonitis are associated with increased perioperative mortality and poor outcome. In cases with perforations proximal to the carcinoma perioperative mortality is increased significantly [5].

## Conclusion

It is very rare to encounter a colonic carcinoma with multiple perforations situated markedly proximal to tumor and multiple in number. Colonic carcinoma with incompetent ileocaecal valve and obstruction may lead to ischemic enteritis and multiple perforations along antimesenteric border of small bowel. Small perforations covered with omentum and without peritoneal contamination leads to reduced perioperative morbidity. Prognosis in such cases is better than carcinoma colon presenting with colonic perforations.

## Competing interests

The author(s) declare that they have no competing interests.

## Authors' contributions

**SKT **designed the study and participated in writing process.

**MKS **carried out bibliographic research and data acquisition and helped to draft the manuscript.

**RK **revised the manuscript.

**AKK **designed the study, drafted and revised the manuscript.

All authors read and approved the final manuscript.

## Funding source

None
